# The Potential for Plant-Based Diets to Promote Health Among Blacks Living in the United States

**DOI:** 10.3390/nu11122915

**Published:** 2019-12-02

**Authors:** Samara R. Sterling, Shelly-Ann Bowen

**Affiliations:** 1 The Peanut Institute 1, Albany, GA 31707, USA; 2 ICF International 2, Atlanta, GA 30329, USA; shelly-ann.bowen@icf.com

**Keywords:** diet patterns, plant-based, vegetarian, vegan, African American, health disparities, chronic disease

## Abstract

Plant-based diets are associated with reduced risks of various chronic diseases in the general population. However, it is unclear how these benefits translate to Blacks living in the United States, who are disproportionately burdened with heart disease, cancer, diabetes, obesity, and chronic kidney disease. The objectives of this study were to: (1) review the general evidence of plant-based diets and health outcomes; (2) discuss how this evidence translates to Blacks following a plant-based diet; and (3) provide recommendations and considerations for future studies in this area. Interestingly, although the evidence supporting plant-based diets in the general population is robust, little research has been done on Blacks specifically. However, the available data suggests that following a plant-based diet may reduce the risk of heart disease and possibly cancer in this population. More research is needed on cardiovascular disease risk factors, cancer subtypes, and other chronic diseases. Further, attention must be given to the unique individual, familial, communal, and environmental needs that Blacks who follow plant-based diets may have. Interventions must be culturally appropriate in order to achieve long-term success, and providing low-cost, flavorful, and nutritious options will be important.

## 1. Introduction

Chronic diseases, such as type 2 diabetes, obesity, stroke, heart disease, and cancer have significant health and economic costs. Currently, 60% of Americans have at least one chronic condition, and 40% have multiple chronic conditions [1]. As the leading contributors of the nation’s $3.3 trillion in annual health care expenditures, chronic illnesses are financially burdensome. Affected patients utilize and spend more on health care services and may have reduced physical and social functioning compared to individuals who are not suffering from chronic diseases [1]. 

Blacks in the United States (people of African ancestry, including African-Americans, Africans, Afro-Caribbeans, and Afro-Latinos) are disproportionately affected with chronic diseases compared to the general population. Several factors contribute to this finding, including genetic predisposition (having high-risk alleles for certain conditions), environmental factors (e.g., poor quality health care), and lifestyle behaviors (diet and physical activity) [2]. A healthy diet and regular physical activity may reduce risks for, delay, and assist in managing, chronic diseases. Fruit and vegetable consumption is a protective factor for cardiovascular disease and a dietary pattern that emphasizes fruits, vegetables, whole grains, legumes, nuts, and seeds may assist in weight management and overall health [3]. Despite the health benefits of increasing fruit and vegetable consumption, only 1 in 10 US adults meet the federal fruit and vegetable recommendations. This is even more of a problem among Blacks, who typically consume lower amounts than Whites [4]. Notably, the diets of Blacks were historically predominantly plant-based in Africa, and elements of that are still seen in some of today’s cultural cuisine [5]. The African Heritage Diet created by Oldways Cultural Foods Tradition emphasizes that the “old ways” of eating consisted mainly of green leafy vegetables, sweet potatoes, fruits, beans, peanuts, coconuts, homemade sauces, herbs, and spices [5]. However, these traditions have shifted, partly through a heavy influence of Western culture, although some remnants of it still remain and commonalities can be seen in the diets of the African diaspora across the Caribbean, Latin America, and the United States [5]. Today, the typical Southern-style diet pattern, as seen in the southeastern region of the United States and among Blacks, is high in fried foods, sweetened beverages, and red and processed meats [6]. Additionally, when compared to more affluent neighborhoods, accessible food outlets in lower-income rural communities where some Blacks reside may not offer fresh fruits and vegetables [7,8,9]. These factors contribute to an unhealthy diet and increased risks for obesity and its related diseases. 

To effectively and equitably address the chronic disease burden, it is important to consider the dietary behaviors of populations most at risk. In this review, we assess the general evidence of plant-based diets and health outcomes. Specifically, we discuss how the data translates to Blacks following a plant-based diet and provide recommendations for future studies designed to either provide further evidence or implement a transition to a plant-based diet. 

## 2. Plant-Based Diets and Their Health Effects

Plant-based diets are dietary patterns that emphasize foods of plant origin, rather than meats and animal byproducts. There is still a degree of heterogeneity in the definition of “plant-based”, since it can describe vegan or vegetarian diets, or semi-vegetarian diets [10,11]. The 2015–2020 USDA Dietary Guidelines describe a “Healthy Vegetarian Eating Pattern” which eliminates meat, poultry, and seafood, and increases intake of legumes, nuts and seeds, and whole grains. Dairy and eggs may also be excluded in a vegan pattern [12]. For the purpose of this review, the terms “plant-based diet” and “plant-based dietary pattern” will include both vegan and vegetarian diets, although reference will be made to predominantly plant-based dietary patterns. 

A 2018 Gallup poll recorded that 5% of Americans identify as vegetarian, and 3% as vegan [13]. Some studies have reported that adherence to a plant-based dietary pattern is associated with lower risks of an array of chronic diseases. Although the dynamics of cultural heritage and genetic characteristics should also be considered, in general, these diets reduce risks for major diseases that disproportionately affect Blacks, such as heart disease, cancer, diabetes, obesity, and chronic kidney disease. Multiple reasons for their benefits exist, including the synergistic effects of nutrients typically found in whole plant foods, such as fruits, vegetables, whole grains, legumes, and nuts [14,15]. Here, we review the current evidence supporting a plant-based diet for disease protection and some of the associated mechanisms.

### 2.1. Heart Disease

Heart disease is the leading cause of death among men and women in the United States and globally [16]. Major risk factors include elevated blood lipids, high blood pressure, and obesity [16]. Currently, Blacks have higher deaths rates from heart disease than other ethnic groups and are disproportionately affected with its risk factors [16]. For example, more than 40% of Black men and women are diagnosed with hypertension, and 82% of Black women are classified as either overweight or obese based on having a body mass index (BMI) of 25 or greater [17,18]. The American Heart Association identifies that reasons for cardiovascular disease disparities in Blacks include physical inactivity, poor quality sleep, cigarette smoking, and poor diet quality (preference for unhealthful “soul food” dishes) [19]. Genetic differences between Blacks and Whites may also play a role, although more cohort studies are needed that identify the prevalence of risk alleles in minority populations, and their relationship to cardiovascular disease risk [19].

Since the emergence of heart disease as a leading cause of death in the early 1900s and its reaching a peak in the 1960s [20], diet has been studied for its potential role. The Diet–Heart hypothesis, coined by Ancel Keys in the 1950s, postulated that saturated fat in the American diet was a major culprit because it increased cholesterol levels [21]. The 2015–2020 Dietary Guidelines for Americans recommends that “individuals should eat as little dietary cholesterol as possible” based on strong evidence showing that “eating patterns that include lower intake of dietary cholesterol are associated with reduced risk of cardiovascular disease” [12]. The document further cautions that “intake of saturated fat should be limited to less than 10 percent of calories per day by replacing them with unsaturated fats” [12]. According to the guidelines, these recommendations are also based on “strong and consistent evidence” showing that substitution of saturated fats with unsaturated fats is associated with reduced risk of both incidences of and mortality from cardiovascular disease [12]. Although the hypothesis continues to be debated, a recent systematic review concluded that replacing saturated fats with poly and mono-unsaturated fats (commonly found in plant sources) is associated with lower coronary heart disease events [22]. Some of the main sources of saturated fat in the U.S. diet include dishes containing cheese, meat, poultry, and seafood [12]. Plant sources of unsaturated fats include olive oil and nuts [12]. Diets that are high in sodium, red meat, and processed meats, and low in nuts/seeds, fruits, and vegetables are also related to increased cardiometabolic death, which may be influenced in part by a higher intake of saturated fats relative to unsaturated fats [23,24]. Although some meats have a lower saturated fat content than others, results from the 2019 APPROACH (Animal and Plant Protein and Cardiovascular Health) trial showed that high intakes of saturated fat were associated with increased cholesterol levels, regardless of meat type (white meat versus red meat) [25]. The lowest cholesterol levels were among non-meat consumers, suggesting that plant-based protein sources may offer the greatest protection from heart disease [25]. Gaush-Ferre et al. found similar results, where substituting red meat with high-quality plant proteins (legumes, soy, nuts) led to more desirable blood lipids and lipoproteins. These results were not seen when substituting red meat for fish or refined carbohydrates [26]. 

In addition to saturated fats, other compounds in meats and animal byproducts have been associated with heart disease. For example, Wang and colleagues demonstrated a two-fold increase in systemic and urine trimethylamine N-oxide (TMAO) levels with chronic red meat consumption compared to white meat or non-meat consumption [27]. Plasma TMAO levels were independent of saturated fat intake. As an atherogenic metabolite, TMAO production may increase the risk of cardiovascular disease in a dose-dependent manner [28]. Wang and colleagues showed that plasma TMAO could be reduced within 4 weeks of discontinuing red meat consumption, thereby potentially lowering heart disease risk [27]. 

Compared to omnivore diets that are high in meats and animal byproducts, plant-based diets are typically lower in saturated fats and cholesterol [29,30]. In addition, several nutrients and compounds found in foods of plant origin like fruits and vegetables are beneficial for the heart. Vitamin K, a fat-soluble vitamin abundant in dark green leafy vegetables, promotes proper blood clotting and protects the arteries [31]. Vitamin K also aids the adequate removal of calcium to prevent calcification in the blood vessels [32]. A recent meta-analysis demonstrated that consumption of leafy green and cruciferous vegetables was associated with a 15.8% reduced incidence of cardiovascular disease in eight studies [33]. Dietary fiber, a non-digestible carbohydrate commonly found in oats, apples, beans, and peas, is also beneficial for the heart in several ways. First, soluble fiber lowers the absorption of cholesterol in the liver by binding bile acids during digestion. This disruption of cholesterol production decreases both total and LDL cholesterol to reduces heart disease risk [34]. High fiber intake also increases satiety, which may promote weight management to reduce the risk of heart disease [35,36]. Heart-healthy mono and polyunsaturated fats, such as those commonly found in avocados, nuts, and olive oil, are favorable for the heart because they help to lower blood pressure and triglyceride levels [22]. 

Additionally, phytochemicals may promote heart health, since many polyphenols decrease endothelial dysfunction to regulate proper heart function. For example, resveratrol, an antioxidant found in the skin of red grapes, works to scavenge free radicals that can cause rupture of plaque along the arterial walls [37], and may reduce risk of hypertension, stroke, and ischemic heart failure [37]. Other polyphenols, such as quercetin and EGCG, induce vascular relaxation to regulate blood pressure and lower the risk of heart disease [38]. 

Plant-based dietary patterns have been shown to promote heart health, although it is unknown if these benefits persist after accounting for genetic variations. For example, Dean Ornish showed in the late 1990s that a low-fat plant-based diet emphasizing fruits, vegetables, and whole grains promoted a regression of coronary atherosclerosis [39]. Plant-based diets that are inclusive of customary levels of fats have also recorded positive results in the management and potential reversal of coronary heart disease [40]. The Portfolio dietary pattern, a plant-based diet created by David Jenkins and colleagues, demonstrated its effectiveness for reducing cholesterol levels after a 4-week intervention. Results were comparable to a very low saturated fat diet that was supplemented with 20 mg of lovastatin, a cholesterol-lowering drug [41]. More recently, the Portfolio diet was associated with a 13% risk reduction of coronary heart disease after 10 years [42]. Thus, the positive association between plant-based diet adherence and heart health is an important finding for the nearly 50% of Black adults who already have some form of heart disease [43]. 

### 2.2. Cancer

Behind heart disease, cancer is the second leading cause of death in the United States [44]. Some modifiable risk factors include low physical activity, poor diet, and obesity [44]. Blacks are disproportionately affected with cancer compared to Whites and generally have lower survival rates [44]. The incidence and death rates for all cancers are higher in Black men than White men (9% and 22%, respectively), and Black women have a 13% higher risk of dying from cancer than White women, even though they have a 7% lower risk of being diagnosed. For specific cancers, the American Cancer Society reports that while the probability of developing breast cancer is higher among White women (13.2%) than Black women (11.5%), Blacks are more likely to die from a diagnosis (3.1%) than Whites (2.6%). Similarly, Black men have a higher probability of developing prostate cancer than White men (14.8% versus 10.6% respectively), and higher death rates than Whites (4.0% versus 2.2%, respectively). Death rates for cancers of the colon and rectum are also higher in Blacks than in Whites (55.2 per 100,000 versus 44.6 per 100,000, respectively) [44]. 

The relationship between poultry/fish consumption and cancer has yielded conflicting results. Some studies have found possible benefits for esophageal squamous cell carcinoma, along with oral cavity and oropharyngeal cancer subtypes with fish consumption [45,46], although more research is needed that considers nutrient variations by fish type [45,46]. For example, the role that omega-3 fats in fish oil play in tumor suppression continues to be investigated, and more interventions are needed on omega-3 dosage and subtypes (DHA versus EPA) [47]. Further, European and Asian cohorts have recorded possible risk reduction of colorectal and breast cancers with omega-3 fish consumption [48,49], but more research is needed in Blacks. Brasky et al. found no significant association between omega-3 and fish consumption and endometrial cancer risk in 47,602 African American women, although non-significant hazard ratios were more favorable among normal weight women (HR = 0.53; 95% CI = 0.18, 1.58) than in overweight women (HR = 0.88; 95% CI = 0.56, 1.31) [50]. 

The World Cancer Research Fund (WCRF) and American Institute for Cancer Research (AICR) both caution that certain fish may increase cancer risk, especially those prepared by salting, smoking, or curing [51]. Salted fish, for example, is associated with increased nasopharyngeal cancer risk [51]. The California Collaborative Prostate Cancer Study observed that pan-frying, oven-broiling, and grilling methods were associated with increased risk of advanced prostate cancer with regular white fish consumption in men (*p* = 0.001) [52]. Today, the most common preparation methods of fish and other meats in Black communities is fried or salted, which may not be associated with risk reduction as in other populations [53,54]. Overall, large prospective studies are needed to further examine the effects of fish intake on cancer risk [55]. Additionally, more evidence is needed on the exposure amounts or specific thresholds where effects may be observed [51].

Other meats have also been linked to cancer. In 2015, the International Agency for Research on Cancer from the World Health Organization published findings on the carcinogenicity of red and processed meat consumption [56]. Processed meats were classified as carcinogenic, increasing colorectal cancer risk by 18% with each 50 g portion consumed daily, and red meat was classified as a probable carcinogen [56]. The 2018 WCRF/AICR report further describes that red meat consumption probably increases colorectal cancer risk by 12% with each 100 g daily portion, and limited evidence was also found for increased nasopharyngeal, lung, and pancreatic cancer risks [51]. More recently, the Nutritional Recommendations (NutriRECS, Halifax, Canada) Consortium challenged the WCRF/AICR recommendations. The Consortium reviewed both randomized controlled trials and cohort studies that were conducted in 1000 or more adults for at least 6 months, and concluded that there was insufficient evidence to suggest that consuming unprocessed red meat and processed meat is detrimental [57]. The recommendation to continue consuming these meats was made based on the view that “the certainty of evidence for the potential adverse health outcomes associated with meat consumption was low to very low, supported by the similar effect estimates for red meat and processed meat consumption from dietary pattern studies as from studies directly addressing red meat and processed meat intake” [58]. However, in response to these findings, Harvard University School of Public Health commented that the methodology used to classify the evidence as “low” or “very low” was inappropriate because of the panel’s use of the GRADE criteria, which is more appropriate for drug trials, instead of the more commonly used HEALM or USDA criteria for large, long-term dietary randomized clinical controlled trials [57]. The University also cautioned that “this recommendation runs contradictory to the large body of evidence indicating higher consumption of red meat—especially processed red meat—is associated with higher risk of type 2 diabetes, cardiovascular disease, certain types of cancers, and premature death” [57]. In a review of 42 published meta-analyses, Lippi et al. identified an increased risk of cancer in consumers of large amounts of red and processed meats, although the risks were not increased for white meat or poultry consumption [59]. More specifically, colorectal, lung, gastric, esophageal, and bladder cancers were identified with increased risks. A recently published longitudinal study of over 42,000 participants identified a 23% increased breast cancer risk with increased red meat consumption [60]. Red/processed meat intake was also positively associated with hepatocarcinomas in men and women in another 2019 study [61].

There are several proposed mechanisms to explain the increase in cancer risk with regular red/processed meat consumption. For processed meats, N-nitroso compounds added during preparation may encourage cancer development endogenously [62]. Steinberg showed a dose-dependent relationship between red meat consumption and nitroso compounds in the feces (249 ± 167 µg nitroso concentration with 420–600 g daily red meat consumption, compared to 54 ± 7 µg nitroso concentration with no daily meat consumption and 87 ± 55 µg with 420–600 g white meat consumption) [63]. Heme iron within red/processed meats can catalyze this reaction, thereby accelerating tumor progression [63]. N-nitrosamines are also found in fried pork, fried bacon, and fried/grilled poultry [64]. Grilled and/or fried meats release heterocyclic amines and polycyclic aromatic hydrocarbons during preparation. Heterocyclic amines have been associated with breast, colorectal, liver, lung, and prostate cancers, while polycyclic aromatic hydrocarbons have been associated with colorectal, breast, and pancreatic tumors [65]. In a recent review of in vivo evidence, Crowe et al. discussed that although most studies report an increased risk of colorectal cancer with consumption of nitrite-containing processed meats, “future epidemiological studies should consider more specifically categorizing the processed meat consumed, explicitly distinguishing between those meats which are nitrite-containing and those which are not” [66]. 

Vegetarians and vegans typically have lower risks of cancer than non-vegetarians [67,68,69,70]. Additionally, there is a lower risk of mortality from breast, lung, and prostate cancers compared to omnivores [67]. Lacto-ovo vegetarian diets may also protect against precursors to cancer, such as gallbladder polyps in adults [71], and regular fruit and vegetable intake is associated with a lower risk of breast fibroadenomas [72]. Plant foods are sources of cancer-protective nutrients and compounds, such as vitamin E, beta-carotene, fiber, and phytochemicals. As an antioxidant, various naturally-occurring forms of vitamin E, including gamma and alpha-tocopherols and tocotrienols, have demonstrated benefits in cancer prevention as well as the potential to be an adjunct therapy for cancer control [73]. Insoluble fibers contained in many fruits and vegetables accelerate the digestive process and facilitate the elimination of carcinogens that come in contact with the intestines [74]. Butyrate, produced through fermentation by the gut microflora, may offer additional protection [74]. 

Plant foods are also abundant in phytochemicals, some of which are thought to stop carcinogenesis through their antioxidant properties by interfering with oxidative stress signaling pathways and suppressing DNA damage [75]. For example, resveratrol seems to reduce estrogen toxicity to protect against breast cancer, and may also reduce the risk of skin, stomach, and pancreatic cancers [76]. β-sitosterol, a phytosterol found in wheat germ, peanuts, and corn oils, may reduce the growth of colon, breast, and prostate cancer cells [77]. The effective phytosterol dosage used in some studies (16 µM) is within typical dietary limits, although the potential effects based on genetic variations and dietary habits of Blacks is not known [77]. Phytosterols may reduce prostate cancer risk by inhibiting tumor growth and stimulating apoptosis [76,77]. Sulforaphane, an antioxidant found in broccoli, has reduced breast tumor size and growth by 75%, and prostate tumor volume by 50% [78]. A 2019 review demonstrated that consuming 500 mg/day of total phytosterols from foods could reduce cancer development risk [79], and a 37% risk reduction was seen for highest dietary phytosterol intake compared to the lowest intake [79]. Phytosterols are highest in vegetable oils (150.4–1230.9 mg/100 g), legumes (129.6–275.6 mg/100 g), and nuts (18.9–255.2 mg/100 g) [80]. 

### 2.3. Diabetes

Type 2 Diabetes (T2D), a chronic disease caused by a reduction in the effectiveness of insulin’s control over blood sugar, is increasing in the United States and globally. Currently, more than 100 million Americans are living with either prediabetes or T2D [81]. Diagnoses for T2D are currently at a rate of 1.5 million per year, and it accounts for close to 80,000 deaths in the United States annually [81]. US Blacks are twice as likely to be diagnosed with T2D as Whites and are also more likely to suffer complications from the disease, such as lower limb amputations and end-stage renal disease [82]. 

Numerous studies have highlighted the role that vegetarian and vegan diets play in reducing the risk of T2D [83,84,85]. Epidemiological studies have documented that vegetarians and vegans have higher insulin sensitivity than omnivores and lower diabetes risk, even after adjusting for BMI [85]. In part, plant-based diets tend to be higher in fiber and lower in saturated fats [29]. High fiber diets increase satiety and lessen a blood glucose spike, thereby decreasing insulin requirements [36], A recent systematic review and meta-analysis noted that dietary cereals, whole grains, and fiber are inversely related to T2D incidence [86]. Recommendations for preventing the disease include weight reduction by diet change and increasing consumption of whole grains, fruits, vegetables, and fiber [86]. These recommendations could provide practical guidelines for patients who are at risk. In addition, reducing intake of animal proteins and increasing intake of plant proteins may play a role. In one study, researchers found that when 5% of energy intake from animal proteins was substituted for plant proteins (peanuts, peanut butter, other nuts, legumes, and whole grains), there was a 23% reduction in risk for T2D [87]. Intervention studies have also confirmed these findings. For example, Lee et al. recorded that A1C improvements were greater for diabetic participants who followed a vegan diet over 12 weeks than for those who did not [88]. 

Diabetes patients are at risk for various neuropathies, which may be influenced in part by an excess of free radicals and oxidative stress [89]. Oxidative stress in other parts of the body can worsen endothelial function and insulin resistance, and may also lead to cellular dysfunction and damage [89]. Various antioxidants found in plant foods have been shown to help to reduce oxidative stress, which can improve nerve and heart function. For example, Vitamins C and E, found abundantly in citrus fruits (vitamin C) and vegetable oils (vitamin E) have both been associated with lower coronary complications, due to their antioxidant properties [90,91]. However, both of these vitamins in large supplemental doses could have adverse effects. The greatest benefit has been observed from dietary doses [90,91]. In a 20 week pilot intervention, participants were randomized to follow either a B12-supplemented low-fat, plant-based (vegan) diet with weekly classes, or their customary diet supplemented with B12. Dietary and clinical data were collected at baseline, midpoint, and at the end of the study. Participants following the vegan diet reported reduced neuropathic pain compared with the control group, as measured by the McGill pain questionnaire (−8.2 point score change, *p* = 0.04), and also reported less neuropathy, as measured by the Michigan Neuropathy Screening Instrument (−1.6 point score change, *p* = 0.03) [92]. 

### 2.4. Obesity

As an independent risk factor for heart disease, cancer, and diabetes, obesity disproportionately affects Blacks, with the highest rates among those living in the southeastern region of the United States (Alabama, 41.8%; Mississippi, 42.9%), and Black women (57.9%) [6,7,8]. In a systematic review and meta-analysis of 86 cross-sectional and 10 prospective studies, the cross-sectional data showed that vegetarians and vegans had significantly lower BMI values than omnivores [67], while another study demonstrated a reduction in waist circumference and fat mass [93]. Evidence suggests that one potential mechanism may be through lowering the concentration of appetite-stimulating hormones, such as leptin. An observational study of healthy volunteers in Poland found that plant-based diets not only lowered body fat storage, but also circulating leptin levels [94]. Since leptin triggers the appetite, lower levels may reduce total caloric intake to benefit weight management. Race was associated with leptin levels in a longitudinal analysis of 62 European American and 58 African-American women, with African-American women having higher circulating leptin levels both at baseline and after weight loss than European American women (baseline: 24.7 versus 19.9 ng/dl, respectively; after weight loss: 11.7 versus 8.48 ng, respectively) [95]. Consistent evidence for the relationship between leptin and obesity is warranted, however, especially in Black populations [95]. In addition to reducing body fat, a plant-based diet may also reduce chronic inflammation associated with the progression of obesity. Following a healthy plant-based diet was shown to reduce chronic inflammation in overweight and obese Iranian women, and even obese vegetarians may have lower adipose tissue inflammation than obese omnivores [96,97]. 

Overweight and obese women have more difficulty conceiving compared with women of normal weight [98]. Obesity increases inflammation and may have a toxic effect on reproductive tissues by promoting cellular damage [98]. Further, changes in hormone regulation that are triggered by obesity can disrupt the ovulatory cycle [99]. Because of this, overweight and obese women are more likely to develop polycystic ovary syndrome (PCOS), which can lead to anovulation [100]. Obese women with PCOS also have higher levels of free testosterone than women of normal weight, which may disrupt fertility [100]. For overweight/obese women and those with PCOS who are trying to conceive, lifestyle interventions that include shifts in dietary patterns are often recommended as the first line of management to promote weight loss [100]. In a clinical trial, women with PCOS who were placed on a vegan diet (39% African-Americans) lost significantly more weight than participants on a regular diet [101]. A cohort study in 711 Indian women found that the shift from a vegetarian dietary pattern to a meat-based pattern with urbanization increased a woman’s risk for PCOS [102]. However, more research is needed on the effects of plant-based diets on PCOS. Riley et al. noted that while there is still little evidence to confirm the role of diet in PCOS, epidemiological and clinical studies point to a possible protective role [103]. Further, since vegetarians tend to weigh less than omnivores, this may reduce some challenges of conceiving. 

### 2.5. Chronic Kidney Disease

Although Blacks represent 13.5% of the US population, they account for more than 35% of all patients receiving dialysis due to chronic kidney disease (CKD) [104]. Plant-based diets have been shown to protect against CKD’s development and progression in multiple studies [105,106,107]. Evaluation of CKD in the Atherosclerosis Risk in Communities (ARIC) study of 14,686 older adults demonstrated that higher adherence to a healthy plant-based diet was associated with lower risk of CKD, as well as slower eGFR decline in participants [108]. Plant-based diets are protective in several ways. First, plant proteins are typically lower in sulfur-based amino acids than animal proteins, which produce more uric acid for kidney filtration [109]. In addition, decreased protein consumption may lower uric acid levels and reduce the risk of acidosis [109]. Fruits and vegetables that are high in potassium help to lower blood pressure, which offers secondary protection against hypertensive-induced nephropathy [109]. For patients with a diagnosis of CKD, ingestion of large amounts of phosphorus (which generally accompanies protein consumption) must be controlled. Plant proteins have a lower bioavailability of phosphorus compared to animal proteins [110]. Approximately 80% of dietary phosphorus is absorbed from animal-based phosphorus, compared with 30–40% absorption from plant-based sources [110]. Additionally, hyperkalemia can adversely affect the nerves, muscles, and heart, and must be closely monitored in CKD patients with an eGFR of <30 mL/min/1.73 m^2^ [111]. Serum potassium levels may be better regulated on a plant-based diet with CKD than on a regular diet since potassium from plants promotes intracellular potassium distribution. Further, high fiber plant-based diets promote increased fecal potassium excretion, thereby preventing elevated potassium levels [109]. Although plant-based diets are typically higher in potassium, they do not promote hyperkalemia in clinical studies [111]. 

## 3. Current Evidence Addressing Plant-Based Diets in Black Populations

Considering the data that exists on the benefits of a plant-based diet for health conditions that commonly affect Blacks, unfortunately, specific evidence that addresses Black populations is limited. The majority of studies are epidemiological in design and have been conducted on a Seventh-day Adventist population, one known for encouraging vegetarianism and healthy lifestyle behaviors among its members [112]. Seventh-day Adventists discourage smoking and drinking alcohol, and many members also limit coffee intake [112]. In addition, for non-vegetarians, consumption of certain meats like pork and shellfish is forbidden [112]. Therefore, while these studies are crucial in order to make public health recommendations, results are limited since they may not be generalizable to various Black populations.

Another epidemiological study, the Reasons for Geographic and Racial Differences in Stroke (REGARDS) study, has also examined dietary patterns and chronic disease risks in 30,239 Black and White participants and offers valuable insight into the connection between diet and disease. Black participants were oversampled, comprising 42% of the study population [6,113]. However, plant-based (vegan/vegetarian) diets were not exclusively examined in this study. Instead, a posteriori dietary patterns were derived using a principal component analysis of 56 different food groups, which identified foods that were customarily consumed as part of specific patterns [6]. As the dietary patterns were identified, a “plant-based” diet emerged, which emphasized vegetables (cruciferous, green-leafy, dark yellow, and other vegetables), fruits, whole grains, legumes, and fish [113]. The inclusion of fish in this pattern makes it difficult to isolate the benefits of an exclusively plant-based diet. Nevertheless, the results of REGARDS offer some insight into how gradually increasing intake of healthy plant-based foods and decreasing intake of animal foods can offer health benefits for Blacks.

### 3.1. Findings in Black Adventists

Much of the evidence of health benefits of plant-based diets recorded in Black Adventist populations pertain to cardiovascular disease and its risk factors (See Table 1). In one of the earliest clinical trials that examined this, Melby et al. noted that, similar to current statistics, US Black adults had a particularly high prevalence of hypertension, possibly due to genetic susceptibility [114]. Results showed that although both non-vegetarian and vegetarian Blacks had blood pressure measurements that were higher than Whites, systolic blood pressure was significantly lower among Black vegetarians when compared to Black non-vegetarians. In fact, 44% of Black non-vegetarians were medicated hypertensives, compared with only 18% of Black vegetarians [114]. Melby and colleagues showed in a later study that Black Adventist vegetarians had significantly lower concentrations of total cholesterol and lower waist-to-hip ratios than non-vegetarians [115]. 

Later research from the Adventist Health Study-2 highlighted additional cardiovascular benefits for Blacks. The study, which enrolled 96,000 Adventists ages 30–112 living in the United States and Canada from 2001–2007, was comprised of 29.6% Black participants. Fraser et al. reported that compared to non-vegetarian Blacks, vegetarian Blacks had lower risks of high cholesterol, obesity, and type 2 diabetes [118]. In another study, lower BMI values were observed in vegetarian Blacks, which promoted longevity compared to non-vegetarians [120]. Interestingly, although Blacks seemed to have a predisposition to Type 2 diabetes, plant-based diets also seemed to be beneficial in this case. Tonstad et al. reported that Black Adventists had a 36% increased risk of Type 2 diabetes compared to non-Black Adventists. However, Black vegans had a 70% reduced diabetes risk and lacto-ovo vegetarians had a 53% reduced risk compared with non-vegetarian Blacks. Results persisted even after controlling for BMI [84]. 

Data from the Adventist Health Study-2 also examined inflammation, which is considered the immune system’s response to irritation or injury. Since it is associated with chronic disease, it is widely believed that chronic inflammation lies at the foundation of, and is probably responsible for, some of today’s most common diseases due to the tissue injury that accompanies it [124]. In a 2012 cross-sectional substudy of the Adventist Health Study-2, Blacks generally had higher levels of C-reactive protein (an inflammatory biomarker associated with cardiovascular disease) than Whites; however, vegetarian diets were associated with lower CRP levels [122]. Inflammation is typically higher in Blacks than in Whites [122,125], and previous research has demonstrated the role that diet plays. For example, diets that emphasize plant foods like nuts, whole grains, fruits, and vegetables are associated with lower inflammation while red meat and dairy may be pro-inflammatory [126]. 

Plant-based diets have also been associated with possible protection from various cancers in Black Adventists; however, more research is needed in this population as well as in the general population. In the Adventist Health Study-2, although Blacks were more likely to adopt pescatarian and non-vegetarian diets than Whites [121], there was a clear association between plant-based diets and the incidence of cancer overall. Vegetarian diets were protective against gastrointestinal cancers and vegan diets were associated with lower overall cancer incidence and female-specific cancers [121]. Separate analyses of prostate, breast, and colorectal cancers showed a trend towards lower risks for Blacks, although the results were non-significant [116,117,119]. 

### 3.2. Findings from the REGARDS Study and Others

The REGARDS study noted an excess in stroke mortality rate among Blacks ages 35–64 and among Blacks living in the southeastern United States compared to Whites, and found that diet may help to offset those risks [127,128]. Among the 21,636 participants who provided dietary information, five main dietary patterns were found: a convenience pattern, a sweets/fat pattern, an alcohol/salads pattern, a “Southern” pattern, and a “plant-based” pattern. US Blacks, particularly Black men and those living in the southeastern portion of the United States, were more likely to adhere to the “Southern” pattern, characterized by a diet high in red/processed meats, fried foods, and sweetened beverages. Meanwhile, older, black females, and those with higher education levels were likely to follow the more plant-based pattern [6]. Judd and colleagues found that adherence to the Southern pattern increased stroke risk by 39% in Black Americans. In contrast, the greatest benefit was seen among participants who followed a more plant-based dietary pattern, which conferred a 29% lower stroke risk [128]. REGARDS data also showed reduced risk for acute coronary heart disease, as well as a 41% lower risk of heart failure with adherence to the plant-based pattern [129,130]. In contrast, the Southern pattern was associated with a 56% increased risk of acute coronary heart disease and a 72% increased risk of heart failure [129,130]. 

Apart from cardiovascular disease, study participants with chronic kidney disease (CKD) who followed a more plant-based pattern had reduced risk of end-stage renal failure (ESRD) mortality, while those who adhered to a Southern pattern experienced a 51% increased risk of ESRD mortality [131]. There were cognitive benefits found with adherence to a mostly plant-based diet in the REGARDS study as well. Greater adherence to the mostly plant-based pattern was associated with higher scores on learning and memory, while the Southern dietary pattern was associated with lower scores [113]. These results coincide with findings of another 2015 study which demonstrated that adherence to a largely plant-based dietary pattern among older Black adults seemed to protect against cognitive decline [132]. 

Following a Southern-style dietary pattern appeared to be even more detrimental for Blacks than for Whites in the REGARDS cohort, which suggests that a shift towards plant-based diets may be particularly useful for this vulnerable population. Since Blacks are often at higher risk for certain conditions, especially chronic diseases, encouraging a transition to plant-based diets could mediate some of those risks. For example, Gutierrez and colleagues described that Blacks were more susceptible to sepsis than Whites, but found that consumption of a Southern pattern put them at an even higher risk than Whites who consumed a similar dietary pattern (Blacks: HR 1.42; Whites: HR 1.21). As with the aforementioned results, a more plant-based diet appeared to decrease risks [133]. The additional benefits conferred upon Blacks following a largely plant-based diet were recorded in a separate cohort study of colorectal cancer and diet in men. Black men who had higher diet scores (indicating a more plant-based diet) had even lower odds of colorectal adenomas than did White men with similar scores [134]. Both of these results suggest that a shift to plant-based diets may offer even greater benefits for Blacks than for Whites. Table 1 describes the findings of plant-based diets in Black Adventists, and Table 2 outlines the main findings in Blacks from REGARDS.

## 4. Nutrition Considerations for Black Adults Following a Plant-Based Diet

According to the official position of the Academy of Nutrition and Dietetics, “appropriately planned vegetarian, including vegan, diets are healthful, nutritionally adequate, and may provide health benefits for the prevention and treatment of certain diseases” [30]. Although the benefits of these diets are well recorded and supported, health professionals may be concerned that recommending them to patients could result in nutritional deficiencies. There are also unique concerns that Blacks could face in adopting a plant-based diet that need to be addressed. It should be remembered there are risks for nutritional deficiencies in any diet, and a plant-based one is no exception. As with any diet, they should be carefully planned. Black adults who follow a plant-based diet should take care to ensure that it is adequate to meet all their nutritional needs. 

### 4.1. Vitamin D

Vitamin D deficiency is more common among Blacks than other ethnic groups. Vitamin D, which is a hormone obtained through sun exposure and diet, tends to be produced in the skin at lower levels in Blacks than in Whites due to darker pigmentation [135]. As a consequence, previous research has shown that even young, healthy Blacks do not receive adequate vitamin D levels at any time of the year [135]. Those who live in Northern regions of the US where sun exposure is minimal in the winter months may have even lower 25 (OH) D concentrations [135]. Low vitamin D status has been linked to higher cancer mortality rates, obesity, diabetes, Alzheimer’s Disease, and osteoporosis [136]. It should be noted, however, that despite lower vitamin D status, Blacks tend to have lower osteoporosis risks than Whites [135,137]. Some studies have found lower vitamin D status among vegetarians compared to omnivores [138,139], while the Adventist Health Study-2 found that although vitamin D values were lower among Black vegetarians than Black non-vegetarians, the result was not significant [140]. Still, because of the potentially increased risks for certain chronic conditions from low vitamin D status, Blacks may need to focus on consuming more plant-based food sources of vitamin D.

Blacks who follow a plant-based diet can get vitamin D from mushrooms and fortified milks. Early evidence suggests that only 12% of Blacks in the US regularly consume mushrooms compared with 20% of Whites [141]. Mushrooms synthesize vitamin D2 with sun exposure, which may then be converted to D3 when ingested [142]. Fortified beverages, such as plant-based milks and orange juices provide approximately 20% of the recommended daily intake for vitamin D [143]. Since early evidence showed that up to 75% of Blacks may be lactose intolerant [144], consuming plant-based milks offers a good alternative to cow’s milk if milk is desired. For those who consume eggs, egg yolks provide a good source of vitamin D, especially from pasture-raised chickens or chickens whose diets have been vitamin D enriched [143].

### 4.2. Vitamin B12

Like other vegetarians/vegans, Blacks who follow a plant-based diet may be at risk for vitamin B12 deficiency. Vitamin B12 is responsible for proper red blood cell formation and neurological function, and is bound to protein in food during digestion [145]. While only small amounts are needed to meet daily requirements, it is still considered an essential nutrient [146]. A deficiency can result in pernicious anemia or disorders of the nervous system, which vegetarians who have lower B12 values than non-vegetarians may have increased risk for [147]. Vitamin B12 deficiency appears to be a risk factor for cardiovascular disease [148], which may be clinically relevant since this chronic disease already affects a large number of Blacks [16].

Since vitamin B12 is mostly found in foods of animal origin, plant-based sources that provide significant amounts of it are usually fortified. A serving of nutritional yeast, which is commonly used in plant-based cheese alternatives, contains three times the recommended intake for vitamin B12 based on the federal recommendations for daily intake (2020), and fortified breakfast cereals can contain over 50% of the amount needed [146]. Other plant-based sources of cobalamin include tea leaves, mushrooms, seaweed, and tempeh, but the amounts provided per serving are not sufficient to meet the daily requirements [147]. Vegetarians who consume eggs or dairy can meet nutrition requirements from either of these sources. One hard-boiled egg contains approximately 0.6 mcg of the 2.3 mcg B12 needed daily, while dairy milk and yogurt supplies approximately 50% of those needs [146]. Plant-based dairy alternatives are often fortified with B12. Therefore, while B12 should be considered in dietary planning, there are a variety of sources on the market that can help Blacks to appropriately meet this nutritional need on a plant-based diet.

### 4.3. Dietary Sodium and Added Sugars

Similarly to other ethnic groups, Blacks tend to consume more sodium than is recommended [149]. This is a consideration that Black vegetarian/vegans should keep in mind. Dietary sodium, for example, is implicated in the development of hypertension, which disproportionately affects Blacks. This may be further complicated by the limited intake of fruits and vegetables that contain potassium, which could lower hypertension risk [150]. It is advised that Blacks consume no more than 1500 mg of sodium per day [149]. This is even lower than the 2300 mg/day recommendation for the general population and for those who are not susceptible to hypertension [151]. Hidden sources of sodium for vegetarians/vegans may come from imitation meats, breads, canned beans, canned vegetables, certain salad dressings, savory snacks, and fermented/preserved foods [149]. To avoid excess sodium intake, Blacks who follow a plant-based diet should be advised to limit their intake to no more than 500 mg per meal in a typical 3-meal day. Limiting the amount of salt added to foods in the cooking process and cutting back on the amount of salt used at the table are also useful tips. 

Additionally, Blacks tend to consume diets that are high in in added sugars, even for those whose dietary patterns are more plant-based [152,153]. Added sugar consumption should be limited to no more than six teaspoons per day [153,154]. Evidence continues to point to the impact that high intakes of added sugars can have on the development of obesity, cardiovascular disease, and overall mortality [152]. Black adults, particularly in the southeast US, consume high amounts of added sugars (more than twice the recommended levels), and Americans get additional sugar from beverages, sweets, grain-based desserts, and dairy desserts [6,152]. Blacks should be careful to limit their sugar intake from these sources in order to increase protection from the aforementioned diseases. 

### 4.4. Healthy versus Unhealthy Plant-Based Diets

A new area of research suggests that not all plant-based diets are created equally in terms of reduced disease risks. For instance, vegans/vegetarians can consume diets that are high in fried/processed foods, convenience foods, and sweets, while still maintaining the technical classification of a vegan or vegetarian. However, the term “plant-based” should focus more on the whole foods that are being included in the diet, rather than on what is being avoided (in this case, meats and possibly animal byproducts). Hu et al. recently noted that the surge of national fast-food chains incorporating plant-based items into their menus may create an illusion of health for concerned customers who are actively seeking to incorporate plant-based meals into their diets [155]. However, theoretically, a veggie burger could contain higher amounts of sodium and the same number of calories as a beef burger [155]. While the substitution of meat for a processed plant-based burger should offer some protection in this case due to its zero cholesterol content, there is not yet sufficient evidence to demonstrate this. As formulations and ingredients of plant-based meat alternatives change, it will be important to examine their nutritional composition to identify potential benefits. 

A 2019 review showed that while healthy plant-based diets reduced cardiovascular disease risk, unhealthy plant-based diets were comparable to animal-based ones which increased risks [156]. The healthy diet was comprised mainly of whole foods like fruits, vegetables, nuts, whole grains, and legumes. In contrast, the unhealthy version was inclusive of refined grains, fried potatoes, and high sugar foods and beverages. A similar study found benefits for cardiovascular disease mortality with healthy plant-based foods, but no such association was found with unhealthy plant-based foods [157]. A recent review emphasized that the type and source of both carbohydrate and fat are important to consider in type 2 diabetes [158]. Unrefined grains are more protective than refined grains, as are poly and mono-unsaturated fats compared to saturated or trans-fats [158]. Another study showed that healthy plant-based diets were associated with lower fatty liver disease risk, while unhealthy plant-based diets might increase risk [159]. A 2019 Harvard study showed that healthy plant-based diets were associated with 0.68 kg less weight gain over 4-year periods, while unhealthy ones are associated with 0.36 kg more weight gain [160]. Therefore, in order to reap the most benefits from a plant-based diet, it should be advised that adherents limit consumption of less healthy foods in favor of minimally-processed whole foods.

## 5. Recommendations for Future Research

First, to address the need for scientific studies on plant-based diets in Black communities, research gaps must be filled. While a plant-based diet will support overall health in Blacks, its potential to address the specific health concerns that Blacks face has not been adequately addressed in the literature. For example, although the evidence for cardiovascular disease protection is strong in Blacks and in the general population, clinical evidence is still needed for cardiovascular disease risk factors. The work conducted by Melby et al. in the 1980s and 1990s regarding hypertension continues to be relevant, yet more up-to-date studies are necessary, considering the observed changes in dietary habits over the years. Food production practices have changed over the past 20 years, and the options available for plant-based diets on the market have diversified. Additionally, food preparation methods in Black communities may be different from other ethnic groups [53,54], and understanding how those methods shift when a person transitions from an omnivorous diet to a plant-based one is crucial. Therefore, research is needed that reflects these changes to ensure that such diets still offer protection for Black adults. Additional studies that target the benefits of plant-based diets for diabetes in Black adults are also needed.

Epidemiological and clinical evidence for other chronic diseases should be presented. For instance, sufficient data on plant-based diets among Blacks and both cancer incidence and mortality are lacking. While the Adventist Health Study-2 found protective trends for breast, prostate, colorectal, and overall cancers in Blacks, some of the results did not achieve statistical significance, and more epidemiological studies in non-Adventist Black populations are needed to confirm these results. This is particularly important because of the high death rates and short survival rates associated with cancer incidence in the Black community. Studies are also needed that focus on less common cancers that affect the Black community, such as kidney, liver, and gastric cancers.

For observational studies, it will be necessary to accurately assess the dietary behaviors. While other ethnic communities may be open and receptive to providing multiple dietary recalls in a research setting, in some Black communities, this could be met with resistance and considered intrusive, which could limit the number of recalls that are collected [161]. Nevertheless, previous studies have found success in using the automated multi-pass method (AMPM) to provide reliable results for dietary intake in Blacks [161]. 

Secondly, research that supports the proper implementation of plant-based diets in Black communities based on the varied individual, familial, environmental, and cultural concerns will be important. Correct implementation will necessitate consideration for culturally palatable foods in order to achieve success. The diet should be centered around healthy foods that are already regularly enjoyed in Black cuisine, such as collards and other dark greens, yams and other tubers, and okra. A cross-sectional study in 410 Black women found that 80% reported eating dark green leafy vegetables in the preceding week, further highlighting the palatability of this food group [162]. Educators may need to teach preparation methods that enhance the nutritive properties of meals, such as eliminating the use of ham hock and other red/processed meats in collard greens and baking breaded vegetables instead of frying them to use less oil. Sautéing methods that eliminate or decrease oil use will also be helpful. James et al. previously showed that one of the barriers to healthy dietary changes in the Black community is the belief that to do so would mean giving up the spices and flavors of cultural foods [163]. Therefore, a variety of herbs, spices, and flavoring vegetables should be used to season plant-based meals so that the richness of flavor is not lost.

In addition to cultural palatability, availability and accessibility must be considered. Evidence-based approaches should be implemented that ensure the long-term sustainability of plant-based diets. For example, for Blacks who live in lower-income or rural neighborhoods, or for those who face food insecurity, strategies on how to adhere to a plant-based diet safely with limited resources are needed. Multi-level strategies (individual and environmental) should also be considered. Early research in this area concluded that “for effective dietary change in African American communities, changes in the food availability will need to precede or take place in parallel with changes recommended to individuals” [164]. Some of our recent work on the nutritional environments in Black Belt rural communities showed that approximately 70% of the accessible food outlets in these neighborhoods were convenience stores, which had a lower availability of healthier food items than grocery stores [165]. Further, the price and quality of healthy foods in both convenience stores and grocery stores were often suboptimal [165]. Therefore, in order for dietary changes to be long-lasting, community and environmental shifts will also be necessary. Community gardens and farmer’s markets may be of great benefit here. One study demonstrated that a farmer’s market’s acceptance of electronic benefit transfer (EBT) for low-income participants (62% Blacks) was related to higher fruit and vegetable consumption [166], and another study showed higher perceived fruit and vegetable intake with access to community gardens in a rural Black community [167], further illustrating the vital role that environments play in dietary behaviors.

There is also a common misconception that plant-based diets are more costly than animal-based ones, so proper education will be needed. A study published in the Journal of Hunger and Environmental Nutrition showed that a vegetarian diet could result in approximately $750 per year in savings [168], and a 2018 findings from the European Association for the Study of Obesity similarly found that vegetarian diets were significantly cheaper than both Mediterranean and standard US diets [169]. Therefore, education will be needed that addresses budget concerns in a way that adequately meets nutrition requirements. Theoretically, a plant-based dinner consisting of red beans, brown rice, collard greens, sweet potato, and cornbread could feed a family of four for under $12 or $3 per person (see Table 3). Such a meal is not only cost-effective, but nutritious, providing protein, fiber, vitamin K, calcium, β-carotene, B vitamins, and a host of antioxidants to protect against various diseases like heart disease and cancer. Likely, the healthcare costs that would be saved with a global shift towards more plant-based diets would apply to Black communities as well. Table 3 outlines the average costs of and key nutrients in select cultural plant foods, along with the health benefits that may be offered from their consumption.

Nutrition interventions geared towards reversing obesity, type 2 diabetes, heart disease, and other conditions are also needed in Black communities. Since disease onset is a powerful motivating factor for dietary and lifestyle changes, it presents an opportunity for scientists to utilize whole food, plant-based diets as an aid in the management and potential reversal of chronic disease. These interventions should not be geared only towards the individual, but towards the family and surrounding community as well. Community-based participatory study designs have achieved success for lifestyle changes in Black populations, mainly because diet and lifestyle are so intricately intertwined with Black culture [170]. Cultural identity is impacted by the foods a person eats, and this can result in peer pressure to conform to the cultural norms. In addition, the diets of family members are often influenced by others in the home. Previous research has shown that regular trips to the grocery store by the primary household shopper may result in increased fruit and vegetable consumption [171]. Therefore, research models that consider the individual and their spheres of influence will be indispensable. 

## 6. Public Health Implications

Poor dietary habits are a global problem. The 2019 Global Burden of Disease study found that most countries consume too few nuts and seeds, and too much sodium and red meat. In fact, dietary intake of all unhealthy foods/nutrients exceeds the optimal level globally. In Latin America, a diet low in nuts and seeds was responsible for the greatest proportion of dietary-related deaths in 2017, while in sub-Saharan Africa, it was a diet low in fruits [187]. Authors conclude that there is an urgent need to improve human diets globally, as this could potentially prevent one in five premature deaths each year [187]. Similarly, the EAT Lancet Report called for a global shift in dietary habits for both health and sustainability reasons. The results of the report advised that globally, we need to shift to more plant-based diets by doubling our consumption of fruits, vegetables, legumes, and nuts, and reducing our consumption of added sugar and red meat by approximately 50% [188]. 

Not only would this globally sustainable diet help to alleviate food insecurity, but it may also contribute to decreasing the chronic disease burden in Blacks [109]. Both the US News and World Report and the Chicago Sun Times reported this year that a growing number of Blacks are transitioning to plant-based diets because of the unique and varied health issues they face [189,190]. This coincides with earlier research done by James and colleagues, highlighting that within the Black community, changes in the diet are often precipitated by a disease diagnosis [163]. This subculture of Black vegans and vegetarians is debunking the common stereotype that plant-based diets are generally palatable for, and thus only practiced by, affluent Whites, or that plant-based meals would be in direct opposition to the “soul food” that Blacks are accustomed to [189,190]. For some adherents, the concept of social justice plays a role, as they seek to reverse the effects of targeted marketing efforts by fast food companies to children in communities of color [190]. Therefore, interventions that seek to normalize and recommend plant-based diets for Blacks may achieve success by addressing the specific individual concerns (disease diagnoses) and community concerns (social justice) that already exist. 

A multipronged approach (Table 4) that includes many sectors systematically working together has the potential to increase the acceptance of plant-based diets. Current evidence demonstrates a need for increased culturally appropriate plant-based cookbooks and materials. Use of community participatory strategies to develop such products will increase acceptance and use of the materials. Simultaneously policies and programs that increase the availability of affordable fruits and vegetables, especially in food deserts will be important for the population to have access. In addition, although this is shifting, plant-based nutrition has not yet been adopted in standard treatment practices by mainstream medicine [191]. Thirty-three percent of physicians surveyed at Rush University Medical Center said they would recommend a plant-based diet to their patients, and while most were aware of some of the health benefits of the diet, they were generally unaware of the details [10]. Clinical provider interventions, such as campaigns to increase provider awareness about the benefits of recommending plant-based diets to their patients, could address patient risk and disease. Finally, enhanced surveillance to monitor fruit vegetable intake behaviors and environments will provide essential data and information to determine the health impact of the changes to a plant-based diet or disease risks and prevention.

## 7. Conclusions

In general, plant-based diets are associated with reduced risks of numerous chronic diseases, including heart disease, diabetes, cancer, obesity, and chronic kidney disease. However, more research is needed on the potential effects for Blacks living in the United States, who are disproportionately burdened with these conditions. The available evidence points to protection from heart disease and possibly cancer for Blacks who follow plant-based diets, but more research is needed for cardiovascular disease risk factors, cancer subtypes, and other chronic diseases. Future research in this area should utilize culturally appropriate methods to increase adherence in the implementation of plant-based diets in Black communities. Multi-level strategies will contribute to the long-term success of these efforts. 

## Figures and Tables

**Table 1:** Main studies of plant-based diets and health in Black Adventist participants.

Year	Author(s)	Health Focus	Outcomes	Reference
2016	Tantamango-Bertley et al.	Prostate cancer	Vegan diet was protective against prostate cancer in non-Blacks (HR ^a^: 0.65; 95% CI ^b^: 0.49, 0.85). There was a similar but non-significant point estimate for Black vegans (HR: 0.69; 95% CI: 0.41, 1.18)	[116]
2016	Penniecook-Sawyers et al.	Breast cancer	Among both Blacks and non-Blacks, a vegan diet was possibly protective against breast cancer compared to a non-vegetarian diet (HR 0.78; CI 0.58, 1.05; *p* = 0.09)	[117]
2015	Fraser et al.	CVD ^c^ risk factors	Compared to non-vegetarian Blacks, vegetarian Blacks had: 52% lower T2D ^d^ risk; 58% lower risk of high total cholesterol; Lower LDL ^e^ cholesterol; Lower obesity rates Vegetarian diets possibly protected Blacks from CVD risk factors	[118]
2015	Olrich et al.	Colorectal cancer	Vegetarians had lower risk of colorectal cancer in non-Blacks (HR 0.80; CI 0.65–0.98; P = 0.04. Estimates for Blacks were similar but non-significant (HR 0.80; CI 0.53–1.20; *p* = 0.28)	[119]
2014	Singh et al.	Obesity & Longevity	Non-obese Blacks lived 6 years longer on average than obese Blacks Vegetarian Blacks had BMI values that were 2–4 kg lower than non-vegetarians Plant-based diets offered protection against obesity	[120]
2013	Tonstad et al.	Type 2 Diabetes	Blacks were more likely to develop T2D than non-Blacks (OR ^f^ 1.364; CI 1.093–1.702) Vegetarian diets were protective against T2D in Blacks	[84]
2013	Tantamango-Bertley et al.	Cancer	Vegetarian diets were protective against overall cancer incidence in Blacks and Whites	[121]
2012	Paalani et al.	Inflammatory markers	Blacks had higher CRP ^g^ and IL-6 ^h^ levels than non-Blacks (β = 0.118; 95% confidence interval = 0.014–0.206; *p* = 0.025) Vegetarian diets were associated with lower CRP levels in Blacks, and may be protective against inflammation	[122]
1994	Melby et al.	Blood pressure; Serum lipids	Black vegetarians lad lower mean waist-to-hip ratios and serum lipids than non-vegetarians	[115]
1993	Melby et al.	Blood pressure	Blacks were more susceptible to HTN ^i^ than Whites Adherence to a long-term vegetarian diet protected Blacks against HTN	[123]
1989	Melby et al.	Blood pressure	Lower systolic blood pressure among Black vegetarians (122.8) compared to Black non-vegetarians (129.7) Black vegetarians had higher systolic blood pressure than Whites	[114]

^a^ Hazard ratio; ^b^ 95% confidence interval; ^c^ cardiovascular disease; ^d^ type 2 diabetes; ^e^ low density lipoprotein; ^f^ odds ratio; ^g^ c-reactive protein; ^h^ interleukin-6; ^i^ hypertension.

**Table 2:** Main findings related to principal component analysis (PCA) derived dietary patterns and health in Blacks from the REGARDS study.

Year	Author(s)	Health Focus	Outcomes	Reference
2019	Lara et al.	Heart failure	PBP ^a^ = 41% lower heart failure risk SDP ^b^ = 72% higher heart failure risk	[130]
2016	Pearson et al.	Cognition	PBP = higher learning and memory scores than SBP	[113]
2016	Akinyemiju et al.	Cancer mortality	PBD decreased cancer mortality risks in Whites, but not in Blacks	
2015	Suwaidi et al.	Acute CHD	SDP = 56% increased risk, while PBP protective	[129]
2015	Gutierrez	Sepsis	SDP = increased sepsis risk, especially in Blacks PBD = reduced sepsis risk, although still higher in Blacks than Whites	
2014	Gutierrez	ESRD mortality	PBD reduced ESRD ^c^ mortality risk SDP increased ESRD mortality risk	[131]
2013	Judd et al.	Stroke	Adherence to a Southern dietary pattern increased stroke risk by 39% Adherence to a PBD was protective against stroke in Blacks (HR ^d^ 0.71; CI ^e^ 0.56–0.91)	[128]

^a^ Denotes a mostly plant-based pattern; ^b^ denotes a Southern-style dietary pattern; ^c^ end-stage renal disease; ^d^ hazard ratio; ^e^ 95% confidence interval.

**Table 3:** Potential health benefits of select plant foods present in Black cuisine.

Food Group	Select Cultural Foods	Approximate Retail Cost USD (2019) [172,173]	Some Key Nutrients and Compounds [174]	Potential Health Benefits
Fruits	Apples, bananas, pears, peaches, papayas	$0.55–$1.89/lb	Vitamin C; fiber; polyphenols; folate; potassium	Brain health; protection from hypertension [175,176]
Dark green leafy vegetables	Collard greens, turnip greens, mustard greens, kale	$0.62–$2.88/lb	Vitamin K, Calcium, Folate, carotenoids, flavonoids	Immune system support; cancer protection [177]
Other vegetables	“Flavoring” vegetables: onion; garlic; leek	approximately $1.05/lb	Organosulfur compounds; flavonols	Immune system support; heart health; cancer protection [178]
	Tomatoes	$2.01/lb	Lycopene; vitamin C; biotin; vitamin K	Prostate cancer protection; heart health [179,180]
	Mushrooms	$3.55/lb	Vitamin D; Selenium Folate; Choline	Cancer protection; immune system support [181]
Roots and tubers	Sweet potatoes; carrots; cassava; breadfruit; plantains	$0.60–1.05/lb	Iron; Fiber; B-vitamins; B-carotene; Potassium; phenolic compounds	Heart health; protection from cancer and type 2 diabetes; antimicrobial properties [182]
Whole grains	Cornmeal/maize; oatmeal; brown rice; whole wheat flour	$0.08–1.28/lb	B-vitamins; fiber; zinc; protein; biotin	Protection from obesity, type-2 diabetes, colorectal and pancreatic cancer [183]
Legumes	Kidney beans; black beans; black-eyed peas; lentils; chickpeas	$1.56–1.69/lb	Protein; folate; iron; zinc; fiber; vitamin E	Reduction of inflammation; type-2 diabetes prevention; heart health [184]
Nuts and seeds	Peanuts; cashews; sunflower seeds	$0.15–0.43/oz	Protein; vitamin E; folate; zinc	Heart health; weight management; cancer protection [185]
Herbs and spices	Ginger; turmeric; cinnamon	N/A	Gingerol; curcumin; cinnamic acid	Protection from inflammation and cancer [186]

**Table 4:** Multi-pronged approach for strategies and interventions focused on plant-based diets for Blacks living in the United States.

Strategies and Interventions	
**Research**	Focus on community-based approaches as a unique opportunity for this group Develop culturally-appropriate cookbooks and other materials Do more research on how to implement this diet long-term in Black communities
**Policies and Programs**	Implement food policy councils to develop policies related to healthy food access, including fruit and vegetable production, availability, and distribution. Menu labeling laws, pricing strategies, limitations for public assistance programs such as SNAP on types of food purchased Implement initiatives to bring supermarkets to underserved areas
**Environmental Approaches**	Provide communities support to increase access to affordable fruits and vegetables in food deserts Work with local supermarkets to stock healthier food items Provide public-service education that debunks the misconception that plant-based diets are expensive/tasteless Consider policies, programs, and initiatives that work with retailers and local farmers to increase the availability of affordable produce in the community
**Healthcare Provider Education**	Increase healthcare providers knowledge about the health benefits of a plant-based diet
**Enhance Surveillance**	Monitor trends and track progress on dietary intake
